# The tumor microenvironment of pancreatic adenocarcinoma and immune checkpoint inhibitor resistance: a perplex relationship

**DOI:** 10.20517/cdr.2020.48

**Published:** 2020-09-04

**Authors:** Irem Sahin, Sevda Turen, Pranav Santapuram, Ibrahim Halil Sahin

**Affiliations:** ^1^Baskent University School of Medicine, Department of Medicine Ankara, Ankara 06810, Turkey.; ^2^TC Istanbul Kültür University, Faculty of Health Sciences, Department of Nursing, Istanbul 34158, Turkey.; ^3^Emory University School of Medicine, Department of Medicine, Atlanta, GA 30322, USA.; ^4^Moffitt Cancer Center, Department of Gastrointestinal Oncology, Tampa, FL 33612, USA.

**Keywords:** Pancreatic adenocarcinoma, durvalumab, nivolumab, ipilimumab, pembrolizumab, immune checkpoint inhibitors, immunotherapy resistance, mismatch repair deficient, microsatellite instability high, microsatellite stable, tumor-associated macrophages, myeloid-derived suppressor cells, T regs, T cells

## Abstract

Pancreatic cancer is one of the most aggressive cancers with a high mortality rate even among patients with early-stage disease. Although recent studies with novel therapeutic approaches have led to modest improvement in survival outcomes, limited progress is achieved for the use of immunotherapeutics in this challenging cancer. Immune checkpoint inhibitors, thus far, single-agent or in combination, have not yielded significant improvement in survival outcomes except in mismatch repair-deficient pancreatic cancer. The tumor microenvironment of pancreatic cancer has been considered as an attractive target for over a decade based on preclinical studies that suggested it may adversely affect drug delivery and antitumor immunity. In this review article, we elaborate on the biology of pancreatic cancer microenvironment, its highly complicated interaction with cancer cells, and the immune system. We also discuss plausible explanations that led to the failure of immune checkpoint inhibitors as therapeutic agents and the potential impacts of pancreatic cancer stroma on these negative studies.

## Introduction

Pancreatic adenocarcinoma is one of the most challenging cancers among solid tumors with limited progress in therapeutic options particularly in advanced-stage disease leading to dismal outcomes^[1]^. Over the last decade, dramatic changes in immunotherapy and targeted therapy resulted in significant change in the horizons of cancer management in the field of cancer therapeutics^[2]^. These novel drug development concepts have led to a dramatic shift in the paradigm of cancer treatment in solid tumors. For example, comprehensive molecular profiling with the next generation of sequencing has evolved significantly and is currently standard of care in advanced stage solid tumors including pancreatic adenocarcinoma^[3,4]^. Upfront analysis of molecular alterations that are in actionable genes provides a framework to guide the therapy for personalized medicine. Notably, clinical trial concepts have also shifted to biomarker-based studies which make molecular profiling an essential tool for clinicians to practice precision medicine.

This progress has also led to the development of novel therapeutic concepts in pancreatic adenocarcinoma as well. Recently, olaparib, a poly(ADP-ribose) polymerases inhibitor, has resulted in improvement in clinical outcomes in BRCA1/2 mutant metastatic pancreatic adenocarcinoma patients when given as maintenance therapy after initial induction cytotoxic treatment^[5]^. Immunotherapy has also achieved some degree of progress, although limited, in pancreatic adenocarcinoma. Pembrolizumab, a humanized immunoglobulin G4 antibody directed against programmed cell death protein 1 (PD-1), has been approved by FDA in mismatch repair deficient (MMR-D) solid tumors, including pancreatic cancer^[6]^. However, the frequency of MMR-D among pancreatic adenocarcinoma patients is approximately 1% and makes this promising therapy available to only a very small subset of pancreatic cancer patients^[7]^. Although significant efforts have been placed for mismatch repair proficient (MMR-P) pancreatic adenocarcinoma patients, immunotherapy, unfortunately, has not achieved significant progress^[8]^. Notably, both vaccine-based and single-agent immune checkpoint inhibitor therapies have led to disappointing results with no improvement in survival outcomes^[9,10]^.

Intrinsic resistance mechanisms leading to ineffective response to immunotherapy in pancreatic adenocarcinoma have been investigated in several preclinical and translational studies; however, the exact mechanism of resistance remains unclear. Relatively low mutation burden (approximately 2-5 mutations per Mb)^[11]^ of pancreatic cancer as compared to highly immunogenic hypermutated tumors (> 50 mutations per Mb)^[12]^ was believed to be the underlying reason for the faint immune response against this aggressive cancer. The tumor microenvironment of pancreatic adenocarcinoma, which is also enriched with immunoregulatory cells and dense stroma with hypothetical physical barrier, was considered to play a role in the failure of immunotherapeutic agents^[13]^.

In this review article, we discuss characteristics of pancreatic cancer microenvironment, studies of immune checkpoint inhibitors in pancreatic adenocarcinoma with dismal outcomes, and elaborate on the impacts of the tumor microenvironment on these disappointing results. We also review possible therapeutic approaches to combat the adverse effects of tumor microenvironment on the anti-cancer immune response.

## Pancreatic cancer microenvironment

The pancreatic cancer microenvironment has been investigated in preclinical and clinical studies broadly. Stellate cells that are highly enriched in pancreatic cancer stroma create one of the unique characteristics of this aggressive disease with increased extracellular matrix and collagen leading to fibrotic tumor stroma^[14]^. Pancreatic stellate cells are a highly heterogeneous population of mesenchymal cells that potentially carry myofibroblastic features and are highly responsive to signaling mediators such as platelet-derived growth factor and transforming growth factor-β (TGF-β)^[15]^. The reactive process in the pancreatic cancer stromal tissue which induces highly dense fibrotic tumor stroma and hypovascular microenvironment is called the stromal desmoplasia^[16]^. Desmoplastic stroma is highly enriched with extracellular collagen and matrix creating poor microvascular circulation. These factors have been attributed to functional barrier formation in the tumor microenvironment.

The signaling cascades that mediate desmoplastic reaction have been investigated in pancreatic cancer extensively. Sonic hedgehog signaling, which is highly preserved embryonic signaling, is one of the driving forces of stromal reaction^[17]^. Increased sonic hedgehog signaling was shown to induce stellate cells and myofibroblasts, leading to their proliferation and subsequent desmoplastic reaction^[17]^. This reaction results in dense stroma formation that further leads to tumor hypoxia and aggressive cancer features. Notably, tumor hypoxia itself appears to be one of the inducers of sonic hedgehog signaling^[18]^. This chain reaction in the tumor microenvironment might have an impact on disease biology. For example, a preclinical study showed that upregulated sonic hedgehog signaling accelerates perineural invasion in pancreatic adenocarcinoma, which is associated with adverse outcomes^[19,20]^. Moreover, the sonic hedgehog pathway was also shown to induce lymphangiogenesis, which endows pancreatic cancer cells with metastatic features^[21]^. Notably, sonic hedgehog induced stellate cells reduce CD8+ T cell infiltration to juxtatumor compartment of tumor stroma and creating a potential mechanism to tumor escape^[22,23]^. Sonic hedgehog-activated stromal cells in the tumor microenvironment also secrete several immune mediators including interleukin (IL)-6 and IL-1β which in turn recruit myeloid-derived suppressor cells (MDSCs) to the tumor microenvironment^[23]^. Collectively, these data indicate that sonic hedgehog signaling may have a direct impact on the molecular biology of pancreatic adenocarcinoma along with immune regulation in the tumor microenvironment.

**Figure 1:**
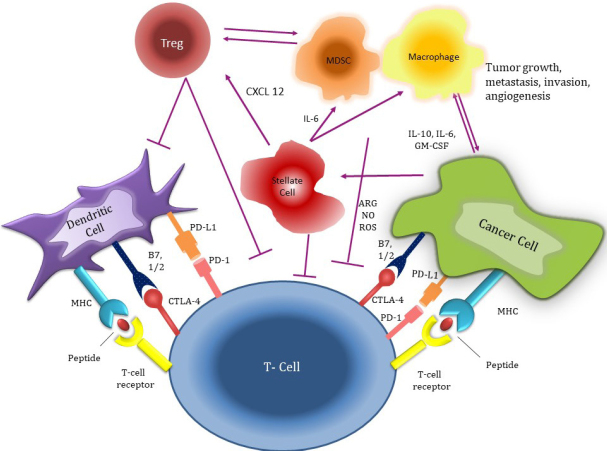
The interaction between pancreatic cancer cells, stroma cells, and immune system. MHC: major histocompatibility complex; PD-L1: programmed death-ligand 1; CTLA4: cytotoxic T-lymphocyte-associated protein 4; CXCL12: C-X-C motif chemokine 12; IL-6: Interleukin 6; GM-CSF: granulocyte-macrophage colony-stimulating factor

The stroma of pancreatic cancer is also highly enriched by immunosuppressor cells. FOXP3+ regulatory T cells (T regs) are found abundantly in the pancreatic cancer microenvironment and they have a direct role in the molecular behavior of cancer cells and their relationship with effector T cells^[24]^. They are known to induce cancer progression and are associated with adverse outcomes in pancreatic cancer^[25]^. Notably, there is also evidence suggesting that depletion of T regs may accelerate the progression of pancreatic cancer^[26]^. T regs directly impair CD8+ effector T cells activity, which results in tumor immune escape in solid tumors^[27]^. For example, an in-vivo study reported increased TGF-β signaling orchestrated by T regs suppresses the cytotoxic effect of CD8 + T cells^[28]^. Toll-like receptor-8 signaling has also been found to be a mediator suppressive function of T regs on cytotoxic T cells, which appears to induce immune tolerance^[29]^. Notably, cytotoxic T-lymphocyte-associated protein 4 (CTLA4) expressing T regs impair the antigen-presenting function of dendritic cells, which in turn leads to inhibition of effector T cells^[30]^. T regs also deplete IL-2 in the tumor microenvironment resulting in abrogated type 1 CD4+ T cell (Th1 cells) function and impaired antitumor response^[27,31]^. There is also limited evidence indicating that T regs may convert ATP to AMP to induce adenosine-mediated effector T cell suppression by inhibiting infiltration of CD8+ T cells to the tumor stroma. MDSCs are also highly prevalent in pancreatic cancer stroma^[32-34]^. MDSCs are recruited to the tumor microenvironment by cancer and tumor stromal cells by using tumor-associated inflammatory mediators^[33,35,36]^. Similar to T regs, MDSCs also result in a diminished adaptive immune response against cancer cells^[37]^. Notably, these immune regulatory cells compete with effector immune cells for cysteine and other essential nutritional elements that are essential for cytotoxic T cell function^[37]^. MDSCs also secrete several interleukins such as IL-10, which upregulates T regs, and type 2 CD4+ T cells (Th2) function, which turns-off effector T cells^[23]^. Tumor-associated macrophages are another group of negative regulators of effector T cell function in pancreatic cancer and the inhibition of these cells results in improved antitumor immune response in preclinical studies^[38]^. Tumor-associated macrophages also recruit regulatory cells particularly T regs by stimulating 15-lipoxygenase-2 pathway^[39]^. A study demonstrated increased expression of chemokine C-C motif ligand 20 (CCL20) by tumor-associated macrophages, which attracts T regs via CCR6 that serves as a receptor for CCL20^[40]^. Collectively, these data suggest that tumor-associated macrophages mediate anticancer immunity by enhancing the T reg function in the pancreatic cancer microenvironment. Notably, there is also growing evidence that there is a cross-talk between tumor-associated macrophages and MDSC via secretion of IL-6, which leads to the increased negative regulatory activity of MDSCs and T regs in tumor stroma^[23]^. These inflammatory cells in pancreatic cancer stroma play an important role in immune dysregulation promoting ineffective immune surveillance and antitumor response [Figure 1].

In conclusion, the data above suggest a highly preserved and sophisticated interaction between cancer and tumor stromal cells and the immune system with multifaceted interaction leading to a unique tumor microenvironment with increased inhibitory signals on effector T cells and thus creating a safe haven for cancer growth in pancreatic cancer. Although these discoveries have enlightened important biological features of pancreatic cancer stroma, at this time, many aspects of this multifaceted communication remain unknown, which at least partially contributed to the failure of clinical trials discussed below.

## Clinical trials investigating immune checkpoint inhibitors in pancreatic adenocarcinoma

Clinical trials have investigated the efficacy of immune checkpoint inhibitors in pancreatic cancers as a single agent and combination models. Ipilimumab, one of the first immune checkpoint inhibitors targeting CTLA4, has been investigated as a single agent in pancreatic adenocarcinoma patients^[41]^. This phase II trial enrolled 27 patients with advanced-stage disease and no objective response was reported by the authors^[41]^. Another immune checkpoint inhibitor targeting programmed death-ligand (PD-L1) (BMS-936559) was investigated in patients with advanced-stage solid tumor and there was no signal in pancreatic cancer patients^[42]^. Pembrolizumab, a humanized anti-PD-1 antibody, has been investigated in multiple solid tumors in phase Ib study, similar to previous experiences and no promising anti-cancer effect was observed^[43]^. Most recently, durvalumab (a human immunoglobulin directed against PD-L1) was investigated with or without tremelimumab (a fully human antibody targeting CTLA4) in metastatic pancreatic adenocarcinoma patients^[10,44]^. In this phase II study, 65 previously treated patients were enrolled. The objective response was noted in only one patient in combination arm (3.1%), and there was no objective response in single agent durvalumab arm, indicating that pancreatic adenocarcinoma has innate resistance to immune checkpoint inhibitors.

Immune checkpoint inhibitors were also investigated in combination with chemotherapeutics. In a phase Ib trial, ipilimumab was combined with gemcitabine and the study showed a median progression-free survival (PFS) of 2.78 months and median overall survival (OS) of 6.90 months, which are highly similar to historical control outcomes of gemcitabine alone^[45]^. Tremelimumab was also combined with single-agent gemcitabine, and the results of this study were also similar to historical controls with a median OS of 7.4 months^[46]^. The study of pembrolizumab in combination with gemcitabine and nab-paclitaxel investigated the efficacy of this triplet regimen in 17 pancreatic adenocarcinoma patients^[47]^. Although the study showed a modest signal in median overall survival outcomes (a median OS of 15 months) compared to historical controls, the primary endpoint, which was a complete response rate of > 15%, was not met. These studies suggest that immune checkpoint inhibitors do not have an additive role in cytotoxic therapies for the treatment of pancreatic adenocarcinoma. The synergistic effect between immune checkpoint inhibitors and chemotherapy that have been noted in other solid tumors such as non-small cell carcinoma^[48]^ appears to be not applicable to pancreatic cancer, suggesting chemotherapy-mediated neoantigen release is not an appealing approach at this juncture.

Immune checkpoint inhibitors were also investigated in combination with cancer vaccines. GVAX, a whole tumor vaccine designed to prime anticancer immunity, was investigated in previously treated metastatic pancreatic adenocarcinoma patients. GVAX in combination with live-attenuated listeria-encoding human mesothelin vaccine (CRS-207) and low dose cyclophosphamide (ECLIPSE trial) was investigated in a phase IIb trial, which failed to show any improvement in survival outcomes as compared to physician’s choice of single-agent chemotherapy^[9]^. This novel vaccine concept is currently being investigated in clinical trials in combination with immune checkpoint inhibitor therapy. In a phase II trial, nivolumab is combined with GVAX and low dose cyclophosphamide (NCT02243371), and the results of this trial will be reported soon. Similarly, pembrolizumab is currently being investigated in combination with GVAX and low dose cyclophosphamide along with SBRT in locally advanced pancreatic adenocarcinoma (NCT02648282). In another clinical trial, the combination of nivolumab, ipilimumab, and CRS-207 as a triplet therapy is being investigated with or without GVAX (NCT03190265). The efficacy of immune checkpoint inhibitors and cancer vaccines is yet to be proven, and these ongoing studies may shed further light on our understanding of the immunogenicity of pancreatic adenocarcinoma.

Pancreatic adenocarcinoma patients with MMR-D carry classical characteristics of MMR-D with high tumor mutation burden and accelerated neoantigen generation, which are highly immunogenic self-proteins created by frameshift mutations^[49]^. Pancreatic cancer patients with MMR-D have also been included in trials investigating immune checkpoint inhibitors therapy^[50]^. These studies showed highly promising and durable treatment responses across all solid tumors^[6,50]^. This led to the approval of pembrolizumab in a disease-agnostic manner across all solid tumors with MMR-D. Most recently, the Keynote 158 trial investigated the efficacy of pembrolizumab in different types of solid tumors (excluding colorectal cancer) with MMR-D, which included 22 pancreatic adenocarcinoma patients. In this study, the authors reported the overall objective response of 34.8% in the overall cohort while the ORR for pancreatic adenocarcinoma was 18.2%^[51]^. Notably, the PFS and OS were shorter in pancreatic adenocarcinoma patients as compared to other solid tumors (2.1 *vs*. 4.1 months and 4.0 *vs*. 23.5 months, respectively). The duration of response was 13.4 months for pancreatic adenocarcinoma patients while this was unreached for the general population.

## Clinical trials targeting tumor stroma in pancreatic adenocarcinoma

The aggressive nature of pancreatic adenocarcinoma and its resistance to systemic treatments were attributed to unique characteristics of the pancreatic cancer stroma in preclinical studies, which led to drug development interest in this field. Vismodegib, a sonic hedgehog inhibitor, was investigated in pancreatic adenocarcinoma patients in combination with gemcitabine in a phase Ib/II study^[52]^. Unfortunately, this study did not result in any improvement in survival outcomes and, notably, also did not enhance the penetrance of gemcitabine to the tumor microenvironment. Saridegib, another sonic hedgehog inhibitor, also resulted in detrimental outcomes in another study with a higher rate of progression of disease when combined with gemcitabine, leading to termination of this phase II trial^[53]^. Most recently, pegylated recombinant human hyaluronidase (PEGPH20) was investigated with chemotherapeutics. The combination of this agent with gemcitabine and nab-paclitaxel led to promising outcomes in a phase II trial (HALO-202 trial), particularly in patients with high hyaluronan levels, and the authors reported improved PFS (HR = 0.51; 95%CI: 0.26-1.00; *P* = 0.048)^[54]^. The phase III trial of this agent (HALO-301), however, did not show any survival benefit with the addition of PEHPH20 to gemcitabine and nab-paclitaxel, leading to discontinuation of this agent from further development^[55]^.

As detailed above, the pancreatic cancer microenvironment is also highly enriched with several types of suppressor immune cells, which are known to important factors for cancer progression^[56]^. Notably, stromal stellate cells, which promote desmoplastic reaction in the tumor environment, also appear to upregulate proinflammatory cytokines such as IL-6 and recruit MDSCs and tumor-associated macrophages^[57]^. This myeloid inflammatory response has been shown to abrogate anticancer immunity^[58]^. To reverse dysregulated immune balance against antitumor immune response, clinical trials have been designed with novel approaches to expand effector T cell infiltration^[27]^. In an early-phase clinical trial that included pancreatic cancer patients, pegylated recombinant human IL-10 induced promising anti-cancer immunity by expanding CD8+ cytotoxic T cell infiltration in the tumor microenvironment and reversing the MDSC-derived negative regulatory effect on effector immune system^[59,60]^. Based on this promising signal, this concept is further investigated in the phase III trial (SEQUOIA trial). This study combined pegylated IL-10 with FOLFOX in previously treated metastatic pancreatic cancer patients and, unfortunately, that did not result in improvement in survival outcomes, leading to further disappointment^[61]^. Notably, the GVAX trial discussed above implemented low dose cyclophosphamide in the vaccination protocol to eliminate undesired inflammation, particularly T regs recruitment, and this did not translate into a clinical benefit as well^[9,62]^. Taken together, thus far, targeting immune suppressor cells in the pancreatic cancer microenvironment has not led to substantial progress in pancreatic cancer field [Table 1].

**Table 1:** Selected recent clinical trials investigating immunotherapy in stroma targeting approaches in microsatellite stable pancreatic cancer

Study	Trial Design and intervention	Number of patients	Results
Le *et al*.^[6]^ (ECLIPSE Trial)	Phase IIb randomized study of GVAX and CRS-207 in previously treated metastatic pancreatic adenocarcinoma patients. Patients were enrolled to three arms, GVAX+CRS-207(A) *vs*. CRS-207 (B) *vs*. chemotherapy(C)	Arm A = 68 pts Arm B = 58 pts Arm C = 43 pts	There were no statistically significant differences in PFS between study arms: (3.7 *vs*. 5.4 *vs*. 4.6 months in arms A, B, and C, respectively)
Catenacci *et al*.^[52]^	Phase Ib/II randomized study of vismodegib in combination with gemcitabine in pancreatic adenocarcinoma patients	Combination Arm: 53 pts Gemcitabine alone: 53 pts	No improvement in PFS and OS. No improvement in drug delivery into the tumor microenvironment
Ramanathan *et al*.^[69]^ (SWOG S1313)	Phase Ib/II randomized study of PEGPH20 in combination with FOLFIRINOX in previously untreated metastatic pancreatic cancer patients	Combination Arm: 56 pts FOLFIRINOX alone: 55 pts	The combination of PEGPH20 and FOLFIRINOX was detrimental compared to FOLFIRINOX alone particularly due to toxicity (7.7 *vs*. 14.4 months respectively)
Hingorani *et al*.^[54]^ (HALO-202)	Phase II randomized study of PEGPH20 in combination with Nab-paclitaxel/gemcitabine in patients with untreated, metastatic pancreatic adenocarcinoma	Combination Arm: 139 pts Nab-paclitaxel/gemcitabine alone: 92 pts	The combination led to statistically significant PFS improvement (HR, 0.73; 95%CI: 0.53-1.00; *P* = 0.049). No OS differences. PFS improvement was more prominent in patients with high HA levels
Tempero *et al*.^[55]^ (HALO-301)	Phase III study randomized study of PEGPH20 in combination with Nab-paclitaxel/gemcitabine in patients with untreated, metastatic pancreatic adenocarcinoma	Combination Arm: 327 pts Nab-paclitaxel/gemcitabine alone: 165 pts	No difference was seen in PFS and OS between intervention and control arm (median PFS was 7.1 *vs*. 7.1 months; HR = 0.97, 95%CI: 0.75-1.26, median OS for PAG *vs*. AG was 11.2 *vs*. 11.5 months (HR = 1.00, 95%CI: 0.80-1.27; *P* = 0.97)
Hecht *et al*.^[61]^	Phase III Study of FOLFOX Alone and with pegilodecakin (pegylated IL-10) as in metastatic pancreatic cancer patients previously treated with gemcitabine-based chemotherapy (SEQUOIA)	Combination Arm:283 pts FOLFOX alone: 165 pts	No improvement in PFS and OS in interventional arm. (mOS 5.8 *vs*. 6.3 months, HR = 1.05; *P* = 0.65 and mPFS 2.1 months in both arms with HR = 0.98 *P* = 0.81)

PFS: Progression-free survival; OS: overall survival; PEGPH20: Peggylated hyaluronidase

## Ongoing studies of immune checkpoint inhibitors with novel approaches

The studies above investigating the targetability of pancreatic cancer stroma have not introduced practice-changing outcomes. Although the resistance to immune checkpoint inhibitors in pancreatic cancer could be at least partially due to tumor microenvironment, stroma depletion/modification in combination with cancer vaccines and cytotoxic agents have not translated into an improvement in survival outcomes of pancreatic cancer patients. Therefore, it is also important to recognize intrinsic factors directly related to pancreatic cancer characteristics, e.g., low tumor mutation burden and limited neoantigen generation may also have significant roles in resistance to immune checkpoint inhibitor therapy^[49,63]^. Notably, patients with MMR-D pancreatic cancer benefit from immune checkpoint inhibitors, although it appears somewhat less impressive compared to in colorectal and endometrial cancer^[51]^. Collectively, these data suggest that the lack of immune checkpoint inhibitor response in MMR proficient pancreatic cancer is perhaps multifactorial, including both intrinsic and tumor stroma related factors. Therefore, novel strategies targeting the intrinsic factors (including the hypoimmunogenic nature of pancreatic cancer) and immunosuppressive microenvironment of pancreatic cancer might be more likely to overcome the resistance to immune checkpoint inhibitor monotherapy.

Currently, new approaches are investigating the efficacy of the combination of immune checkpoint inhibitors and tumor microenvironment modification. Currently, the KEYNOTE 599 study is investigating the combination of a C-X-C motif chemokine 12 (CXCL12) inhibitor and pembrolizumab, intending to eliminate the negative immune regulatory effect of tumor microenvironment, which is highly enriched with CXCL12^[64]^ (NCT03168139). The preliminary results of this study suggest increased T helper 1-like signature in the tumor microenvironment^[65]^. Currently, phase II trials are also investigating a similar concept with a C-X-C chemokine receptor type 4 (CXCR4) inhibitor and pembrolizumab in metastatic pancreatic cancer patients in different clinical settings with or without chemotherapy (NCT02907099). However, a recent phase II trial with a novel CXCR4 inhibitor in combination with pembrolizumab did not reveal promising outcomes, and only one patient (3.4%) achieved partial response without chemotherapy backbone^[66]^. Colony-stimulating factor1-receptor (CSF-1R) signaling, which promotes macrophage recruitment to tumor microenvironment to fuel cancer growth, is also being targeted in a clinical trial in combination with pembrolizumab and GVAX to reverse the inhibitory signals in patients with borderline resectable pancreatic cancer (NCT03153410). Cabiralizumab (a CSF-1R signaling inhibitor), however, did not result in improvement in outcomes in a phase II clinical trial when combined with nivolumab and chemotherapy in metastatic pancreatic cancer patients^[67]^. Currently, clinical trials are also investigating approaches targeting the myeloid checkpoint, CD47, and early results show promising results for the future^[68]^. Stroma targeting concepts to prime the tumor microenvironment are also being investigated for nivolumab in pancreatic adenocarcinoma patients (NCT03519308). These studies will further enlighten the role of tumor stroma and stroma-driven immune regulatory cells in immune checkpoint inhibitor resistance [Table 2].

**Table 2:** Selected ongoing clinical trials investigating immunotherapy in combination with stroma targeting approaches in pancreatic cancer

Identifier	Trial design	Rationale/phase of trial/current status	Study group
NCT03168139	Olaptesed (CXCL12 inhibitor) alone and in combination with pembrolizumab in pancreatic and colorectal cancer (Keynote-559)	Enhancing the immune response by inhibiting the tumor stroma and cancer cells communication by inhibition of stromal-derived factor 1 (SDF1 also known as CXCL12)	Previously treated pancreatic and colorectal cancer patients
NCT02907099	Pembrolizumab in combination with CXCR4 antagonist in patients with metastatic pancreatic cancer	Enhancing the immune response by suppression of the tumor stroma and cancer cells communication by inhibition of CXCR4 which is a receptor for stromal-derived factor	Previously treated pancreatic cancer patients
NCT03153410	Pembrolizumab in combination with GVAX, and IMC-CS4 (a CSF1-R inhibitor) in patients with borderline resectable adenocarcinoma of the pancreas	Enhancing immune infiltration to the tumor microenvironment by cancer vaccine and inhibition of tumor-associated CSF1-R positive macrophage	Borderline resectable pancreatic adenocarcinoma
NCT03336216	Perioperative chemotherapy and nivolumab in combination with paricalcitol to target the microenvironment in resectable pancreatic cancer	Modulate tumor stroma driven by satellite cells in pancreatic cancer and induce the antitumor immune response	Resectable pancreatic adenocarcinoma

CXCL12: C-X-C motif chemokine 12; CXCR4: C-X-C chemokine receptor type 4; CSF1-R: colony stimulating factor 1-receptor

## Conclusion

In summary, the studies above have led to significant disappointment in the field of pancreatic cancer research. An increasing number of negative studies investigating agents targeting tumor stroma as well as immune checkpoint inhibitors suggests that our knowledge about the tumor microenvironment of pancreatic cancer and its complex relationship with immune cells is highly limited. It is perhaps these gaps in knowledge that have led to a multitude of negative studies. The current fundamental hypothesis suggesting dense stroma-physical barrier is unable to explain this highly complex relationship and the failure of immune checkpoint inhibitor therapy. The hypoimmunogenic nature of pancreatic cancer with low tumor mutation burden at least partially contributes to the de novo resistance to these novel agents. Perhaps the promising responses with the use of immune checkpoint inhibitors seen in MMR-D pancreatic cancer patients indicate that low tumor mutation burden in MMR proficient pancreatic cancer is one of the underlying reasons for the insufficient response to immune checkpoint inhibitor therapy. Further translational studies to better characterize the expression of immune checkpoint protein expression levels, as well as other immune regulatory signals in the tumor microenvironment, are warranted to better understand this highly convoluted relationship. Therefore, while these disappointments have led to diminished enthusiasm for the approaches depleting dense stroma, they also bring with them an opportunity to better understand the relationship among cancer cells, stromal cells, and the immune system to create more precise therapeutic concepts to aggregate anticancer immunity for the future management of pancreatic cancer.

## Declarations

### Copyright

© The Author(s) 2020.
